# Health without Medicine

**Published:** 1889-07

**Authors:** Theodore N. Mead


### HEALTH WITHOUT MEDICINE.

BY THEODORE N. MEAD.

### No. 1.

"Will they never stop tolling that bell?" I said at last aloud and sat up in bed. It was broad daylight, but that is the case in midsummer, even on the south coast of England for about twenty hours out of the twenty-four, so pushing back the chintz curtains which hung in English fashion from a canopy at the bedhead, I got out my watch from under the pillow. It was not yet three o'clock. Scarcely three hours had I enjoyed of much needed and longed for sleep and to be roused up in this hopeless manner was discouraging indeed. Still the bell continued its steady beating. What church in Brighton could be calling to service at this hour of the night in such an importunate way? Finally I arose and going to the oriel window, looked up and down the street. It lay in all the stillness if not in the shadow of night. Not a living creature was to be seen or heard in it, nor in the churchyard opposite, where the old church of grey flints among its yews and elms looked as if it must have stood from time immemorial. But though the sashes were wide open, the sound of the ringing was no louder, to my bewilderment, and did not appear to come from any particular direction but seemed to be in the air. Struck with a sudden thought I laid my finger on my pulse—the beats coincided with the clang of the bell—the tolling was in my own head. My heart sank and I went back to bed once more to wait with open eyes as many a time before, for the slow hours to pass and day to begin.

At breakfast I acceded at last to my host's wish to send for his own physician—with regret and reluctance it must be confessed, for it had been with the hope of escaping from professional treatment that I had gone across the water—and it was not long before the gentleman in question made his appearance in the pleasant drawing-room. He was lean, muscular, red-bearded—this English doctor—and plain spoken beyond anything to which the ears of American patients are accustomed. I told him of my sleeplessness and loss of strength and shortness of breath, and how two different sorts of headaches which had tormented me at intervals for many years had now joined forces with a new and formidable ally, a heavy, dull pain in the back of the head, and among them had obliged me to give up my business and left me little peace day or night. Doubtless to his experienced eye my sunken cheek and lack lustre eye spoke as unmistakably as my words. He inquired about my habits of life and work.

"Yaas," he said, "I understand, you Americans think that it pays to burn the candle at both ends. You have been trying like the rest of your countrymen to do two men's work, and this is the natural result. English moderation pays better than Yankee goaheaditiveness. You get on pretty fast for a time, but you can't *stay* with us. You are worn out at an age when we are in our prime. Now it is my duty to tell you that if you continue your course as heretofore your life will be a short one—indeed if you keep on as you are going just now, a very short one and *no medicine will do you any good.* If all the doctors in the United States tell you anything different don't you believe them, for its all rubbish. But you can save your life yourself in my opinion if you choose to take the trouble."

"Well, Doctor, skin for skin, 'all that a man hath will he give for his life,' says Job, and I am quite of his mind ; now what do you wish me to do?"

"Well, in the first place you must take a great deal more exercise and give up every mental exertion not absolutely unavoidable."

"But, Doctor," I expostulated, "you don't know how much exercise I have been in the habit of taking, a great deal more than most Americans."

"That makes no difference, you have not taken half enough, and what you have taken has not been of the right sort. Your regular daily exercise should be severe enough to send the blood to the brain. Your brain has not been properly nourished and there are symptoms of considerable irritation at the base of it, but in my opinion the difficulty has not yet become chronic. You can never do again, however, the work you have done in the past, that you must make up your mind to. You must live the life of the English country gentleman ; spend as much of your time as you can in the open air, do as little thinking as possible and no reading at all, and twice every day take exercise especially of the upper part of the body that will *send the blood to the brain.*"

These words "Send the blood to the brain," repeated with so much emphasis, struck me with the force of a revelation. Something told me that therein was the key to the whole matter and I resolved to follow the advice, but how to begin was the problem.

"You are fond of riding, get a saddle horse and go up on the Downs," said the doctor.

"But can my head bear the shaking? I have had to give up omnibuses; even walking is painful to me. Almost all the way from Liverpool to London I stood up, and that on my toes, in the railway carriage because the jarring otherwise was too distressing to be endured."

"You have got to fight this thing through," said he. "If you do not conquer it, it will conquer you."

"Then those cold winds on the Downs make my head ache worse and chill me through even in my full winter clothing."

"That is only neuralgic," said he. "It is true it shows a very depressed condition of the nervous system, but if there were nothing the matter with you worse than that, it would be all plain sailing. It won't do for you to go and sit in the sun as you have been doing; you must take other means of getting warm. You must seek relief from such of your headaches as are neuralgic by deadening by the use of cold water the excessive sensibility of the superficial nerves and must warm yourself up by getting the blood in active circulation."

All this is in brief the substance of several consultations during two or three weeks in the course of which I essayed to put in practice the worthy doctor's advice.

Brighton is a charming sojourn for the American who will give him self up to the pleasures and influences of the place without the national restless effort to improve and inform the mind. There are no ruins, no cathedrals, no fine buildings, no historical associations, except the unsavory memories of George the Fourth when Prince of Wales, which still hang about the Pavilion; but the steep and crooked streets have an agreeable quaintness with their little brick and stuccoed houses bright with flowers; from the higher portions the view is most picturesque of the gray town and the blue restless channel; the shops along "the front" that are on the wide and handsome street which runs along the water, fronting the broad promenade bounded by the sea wall—have an air of taste uncommon in England, and rather French than British; the very throngs of visitors, who raise the population up from eighty thousand to a hundred and twenty thousand in the season, add a cheerful vivacity to the scene; while the broad, shingly beach below the sea wall, with its multitudinous life and its fleets of oddly rigged boats, is a place I never tired of. Then there are quaint and picturesque villages to visit, with their little old churches standing all day long with open doors, and behind the town the South Downs, where one can ride for miles—as it were, up against the sky—over the long green swells of turf, and often, far as the eye can reach, see not a house or fence or tree or even shrub, and not a living creature except the rooks and gulls and perhaps a shepherd with dog and flock. So I walked the town and drove with my friends over the beautiful roads as smooth as a floor, and rode a capital little bay hunter, though with a pang at every step, over the aforesaid Downs, and subscribed to the excellent gymnasium and bought tickets for the Royal Tepid Swimming Baths, and in short over did the matter so thoroughly that I was presently laid snugly up within doors. It was discouraging, but the faith was strong within me that though my undue haste had got me a tumble, I had found and still held the clue which would bring me safe out of my labyrinth of troubles, and I presently returned to my American home intending, if necessary, to quit business altogether and bend all my energies to regaining my health.

About three years have elapsed since that memorable visit to Brighton, and instead of abandoning business I am able to give it, without fatigue, as much time as necessary. Instead of lying awake half the night, or more, after an hour's reading, I can study or work till eleven o'clock, sleep like a top and wake refreshed in the morning. I can eat what I like, always in moderation. I have gained thirty pounds, nearly all solid muscle; furthermore, and this is a detail which will interest valetudinarians, the action of my heart, which had become alarmingly weak, has gained strength, and there is a perceptible and considerable increase in the capacity of the lungs. As for headache, or any other ache, I do not have one once a month. Such a result in the case of a man already well advanced in life, is certainly remarkable, and there must be many who feel themselves, or fear themselves, to be, physically, on the downward road, but know not how to turn back, who would be glad to hear how it was accomplished, and who will be encouraged to learn that it was the work of nothing more miraculous then patience and common sense. The *cure* or treatment has consisted simply of *exercise, cold bathing* and *abstinence from drugs* and *stimulants*, and if we Americans could only fully appreciate the value of these simple and inexpensive agencies, it would be of incalculable advantage to the national health and well being, mental, moral and physical. To men and women, to all ages, to all classes, they would be of equal value. In the case of persons in feeble health, indeed these agencies, natural and harmless as they appear, must be made use of with care and judgment, and if the reader will pardon the apparent egotism, I will continue to use the first person and to give my own personal experience as the best means of bringing the matter home to him as one in which not only he should be interested, but in which he, perhaps, has actually a duty to perform to himself, or to others.

(*To be Continued.*)

